# Leucine zipper motif in RRS1 is crucial for the regulation of *Arabidopsis* dual resistance protein complex RPS4/RRS1

**DOI:** 10.1038/srep18702

**Published:** 2016-01-11

**Authors:** Mari Narusaka, Kazuhiro Toyoda, Tomonori Shiraishi, Satoshi Iuchi, Yoshitaka Takano, Ken Shirasu, Yoshihiro Narusaka

**Affiliations:** 1 Research Institute for Biological Sciences Okayama, Okayama 716-1241, Japan; 2 Faculty of Agriculture, Okayama University, Okayama 700-8530, Japan; 3 RIKEN BioResource Centre, Tsukuba 305-0074, Japan; 4 Graduate School of Agriculture, Kyoto University, Kyoto 606-8502, Japan; 5 RIKEN Centre for Sustainable Resource Science, Yokohama 230-0045, Japan

## Abstract

*Arabidopsis thaliana* leucine-rich repeat-containing (NLR) proteins RPS4 and RRS1, known as dual resistance proteins, confer resistance to multiple pathogen isolates, such as the bacterial pathogens *Pseudomonas syringae* and *Ralstonia solanacearum* and the fungal pathogen *Colletotrichum higginsianum*. RPS4 is a typical Toll/interleukin 1 Receptor (TIR)-type NLR, whereas RRS1 is an atypical TIR-NLR that contains a leucine zipper (LZ) motif and a C-terminal WRKY domain. *RPS4* and *RRS1* are localised near each other in a head-to-head orientation. In this study, direct mutagenesis of the C-terminal LZ motif in RRS1 caused an autoimmune response and stunting in the mutant. Co-immunoprecipitation analysis indicated that full-length RPS4 and RRS1 are physically associated with one another. Furthermore, virus-induced gene silencing experiments showed that hypersensitive-like cell death triggered by RPS4/LZ motif-mutated RRS1 depends on EDS1. In conclusion, we suggest that the RRS1-LZ motif is crucial for the regulation of the RPS4/RRS1 complex.

In both plants and animals, intracellular immune receptors of the nucleotide-binding domain and leucine-rich repeat (NLR) protein superfamily play an important role in pathogen recognition and effective innate immune responses^1^,^2^. Plant NLRs use either a direct or an indirect mode for pathogen effector recognition; they either directly bind the cognate effectors or detect changes in host proteins caused by the cognate effectors and subsequently trigger plant defence responses. These modes are known as effector triggered immunity (ETI), which is usually associated with a hypersensitive reaction that often includes localised cell death^3^.

Plant NLRs generally consist of a C-terminal leucine-rich repeat (LRR) domain and a central nucleotide-binding adaptor shared with Apaf-1, plant resistance proteins, and CED-4 (NB-ARC)^3^. NLRs are diverse in their N-terminal structures and possess either a Toll/interleukin 1 receptor (TIR) domain or a coiled-coil (CC) domain^4^. As highly polymorphic and variable parts of plant NLRs^5^, the LRR domains are involved in protein–protein interaction and play a key role in effector recognition specificity^6^,^7^. In contrast, the NB-ARC domain is highly conserved among the majority of plant NLRs. When NLRs are inactive, the NB-ARC domain physically interacts with the LRR domain^8^,^9^,^10^. After effector recognition, the conformational change allows nucleotide exchanges, the replacement of ADP by ATP in the NB domain, and subsequent NLR activation^11^.

Both the N-terminal TIR and CC domains of plant NLRs are considered directly connected to downstream defence signalling components. Overexpression of the TIR and CC domains from plant NLRs causes effector-independent hypersensitive (HR) cell death^12^,^13^. Crystal structure analysis of flax L6 and barley MLA10 also showed that the homodimerisation of the TIR and CC domains is necessary for downstream defence signalling activity^12^,^13^. In addition, the N-terminal domains of plant NLRs may also participate in effector recognition.

Plant NLRs are capable of self-association in the pre- or post-activation state of NLR complexes^1^. However, some plant NLRs require a second NLR for defence signalling^14^, and they form heterodimers or hetero-oligomers. Recent reports indicated that some NLR pairs are required for full disease resistance^14^,^15^. In our previous studies, *Arabidopsis thaliana* NLRs RPS4 (Resistance to *Pseudomonas syringae* 4) and RRS1 (Resistance to *Ralstonia solanacearum* 1), known as dual resistance proteins, confer resistance to multiple pathogen isolates, such as the bacterial pathogens *P. syringae* and *R. solanacearum* and the fungal pathogen *Colletotrichum higginsianum*^16^. RPS4 is a typical TIR-type NLR, whereas RRS1 is an atypical TIR-NLR that contains a leucine zipper (LZ) motif and a C-terminal WRKY domain. *RPS4* and *RRS1* are localised in near each other in a head-to-head orientation. This NLR pair is required to recognise the AvrRps4 effector protein from *P. syringae*, the PopP2 effector protein from *R. solanacearum*, and one or more unidentified effectors from *C. higginsianum*. We recently demonstrated that the introduction of both *RPS4* and *RRS1* confers resistance to multiple, taxonomically distinct pathogen families^17^,^18^. The successful transfer of the *R* gene pair to some plant families (i.e., Brassicaceae, Solanaceae, and Cucurbitaceae) implies that the downstream components of the NLR pair are highly conserved.

The precise mechanism of how RPS4 functions with RRS1 is not clear. Both RPS4 and RRS1 are partially localised in the nucleus of living plant cells in the presence of effectors^19^,^20^. The two proteins likely form a heterodimer and assemble into a complex. Previous studies suggested that RPS4-TIR physically interacts with RRS1-TIR^21^ and the RPS4/RRS1 complex enables the perception of pathogen effectors, AvrRps4 and PopP2, which target the RRS1 WRKY domain and activate defence responses^22^,^23^. RPS4 is also physically associated with EDS1^24^,^25^. This TIR-domain heterodimerisation plays a role in the effector recognition complex consisting of RPS4/RRS1. The objectives of this study were to investigate whether: 1) the full-length RPS4 is physically associated with RRS1, and 2) the RRS1-LZ motif plays a role in the regulation of RPS4/RRS1.

## Results

### Transient expression of dual R proteins

**Figure 1:**
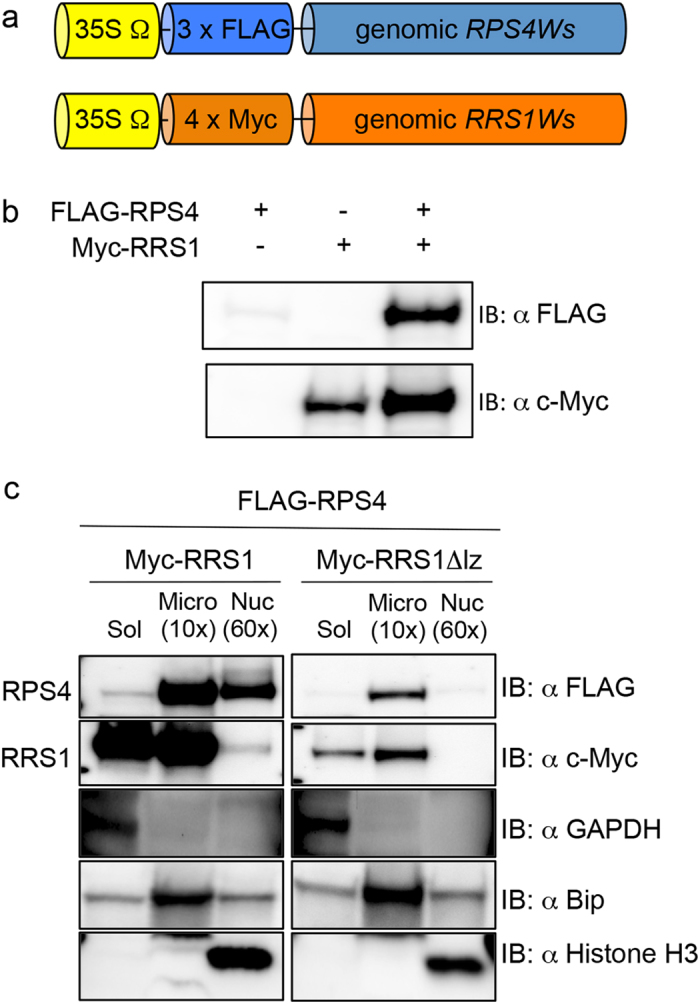
Transient expression of dual R proteins. (**a**) Epitope-tagged constructs used in the transient expression. (**b**) Immunodetection of microsomal extracts from *Nicotiana benthamiana* leaves transiently expressed FLAG-RPS4 and/or Myc-RRS1 44 h after *Agrobacterium* infiltration. (**c**) Subcellular localisation of FLAG-RPS4, Myc-RRS1, and Myc-RRS1Δlz with immunoblotting. Transiently co-expressed FLAG-RPS4 and Myc-RRS1 or Myc-RRS1Δlz mainly localised in microsomal fractions 44 h after *Agrobacterium* infiltration. Microsomal (Micro) and nuclear (Nucl) fractions are 10- and 60-fold more concentrated, respectively, compared with the soluble (Sol) fraction. The degree of fraction enrichment was determined using antibodies against marker proteins (cytoplasmic soluble; anti-GAPDH, microsomes; anti BiP, and nucleus; anti-Histone H3).

To investigate the expression of dual R proteins, RPS4 and RRS1, in *Nicotiana benthamiana* leaves using *Agrobacterium*-mediated transient expression assays, 3 × FLAG and 4 × Myc tags were fused into the N-terminus of RPS4 and RRS1, respectively. These tagged constructs were driven by the cauliflower mosaic virus 35S promoter, while the omega leader sequence, which is known to act as a translational enhancer in plants, served as the 5′ UTR^26^,^27^ (Fig. 1a).

Immunoblot assays of transiently expressed or co-expressed FLAG-RPS4 and Myc-RRS1 in *N. benthamiana* showed that co-expressed full-length RPS4 and RRS1 gave a strong signal on immunoblot compared with transiently expressed RRS1 or RPS4 that were both weakly detected (Fig. 1b). In addition, transiently co-expressed FLAG-RPS4 and Myc-RRS1 were mainly localised in *N. benthamiana* microsomal fractions and weakly localised in nucleus fractions (Fig. 1c). Several previous studies also showed that RPS4 was localised in the microsomal fraction^25^,^28^ and that when transiently expressed in *N. benthamiana*, RPS4 and SNC1 formed a common protein complex in cytoplasmic microsomal compartments^28^.

**Figure 2:**
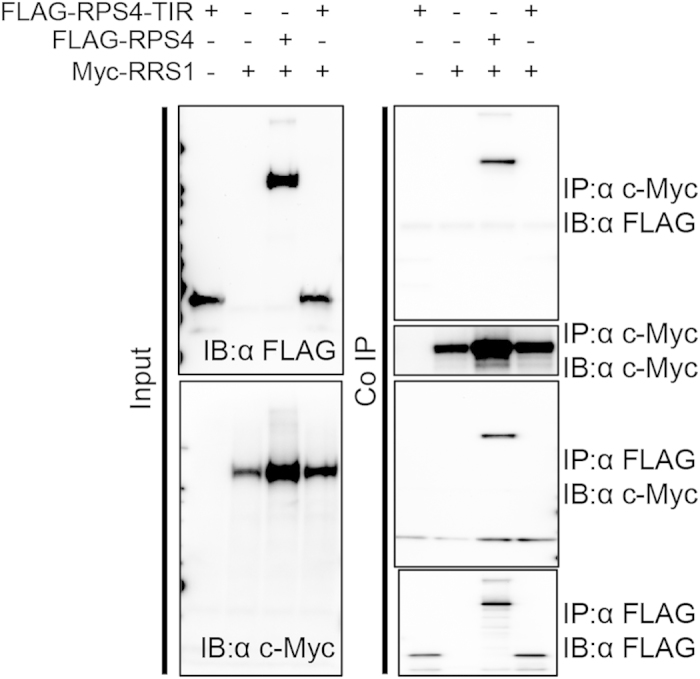
Co-immunoprecipitation analysis of RPS4 and RRS1. RPS4 and RRS1 were transiently expressed in *Nicotiana benthamiana* leaves induced by *Agrobacterium* infiltration for 44 h. Immunoprecipitation (IP) analyses of full-length or TIR domain RPS4 and full-length RRS1 were performed in microsomal fractions with the indicated antibody. The immunoprecipitates were immunoblotted (IB) with the indicated antibody.

Co-immunoprecipitation (Co-IP) analysis on full-length RPS4 and RRS1 proteins isolated 44 h after *Agrobacterium* infiltration showed that they interacted with one another in the microsomal fraction (Fig. 2). The interaction between the RPS4 TIR domain and full-length RRS1 was not detected in the microsomal fraction with Co-IP, although both were detected in this fraction with immunoblotting. Our results suggested that other domains were also required for RPS4/RRS1 interaction, even though the TIR domains of RPS4 and RRS1 formed a heterodimer^21^.

To test whether tag-fused RPS4 and RRS1 confer resistance against *C. higginsianum*, we complementarily introduced N- or C-terminal HA-tagged RRS1 and N-terminal Myc-tagged RRS1 into the *rrs1-1 A. thaliana* Ws-2 accession mutant line. Resistance to *C. higginsianum* was fully restored in the transgenic plants with N-terminal tagged RRS1, but was not restored in the transgenic plants with C-terminal tagged RRS1 (Fig. S1). These results suggested that a proper C-terminus structure in RRS1 is important for *C. higginsianum* resistance in *Arabidopsis*. Similarly, we complementarily introduced N-terminal FLAG-tagged RPS4 and C-terminal YFP-tagged RPS4 into the *rps4-21 A. thaliana* Ws-2 accession mutant line. Resistance to *C. higginsianum* was fully restored in the transgenic plants with N-terminal tagged RPS4, but was not restored in the transgenic plants with C-terminal tagged RPS4 (Fig. S1). Thus, a proper C-terminus structure in RPS4 is likely also important for *C. higginsianum* resistance in *Arabidopsis*. In addition, we showed via immunoblotting that N-terminal tagged 4 × Myc-RRS1 and 3 × FLAG-RPS4 were detected in transgenic *Arabidopsis*, but the transgenic plants with C-terminal tagged RRS1-3 × HA, RPS4-YFP and N-terminal tagged 3 × HA-RRS1 were not detected (Fig. S1). The amount of these proteins may be undetectable with immunoblotting under our conditions. It is important that the N-terminal tagged RRS1 and RPS4 used are functional.

### Mutations in the leucine zipper motif of RRS1

**Figure 3:**
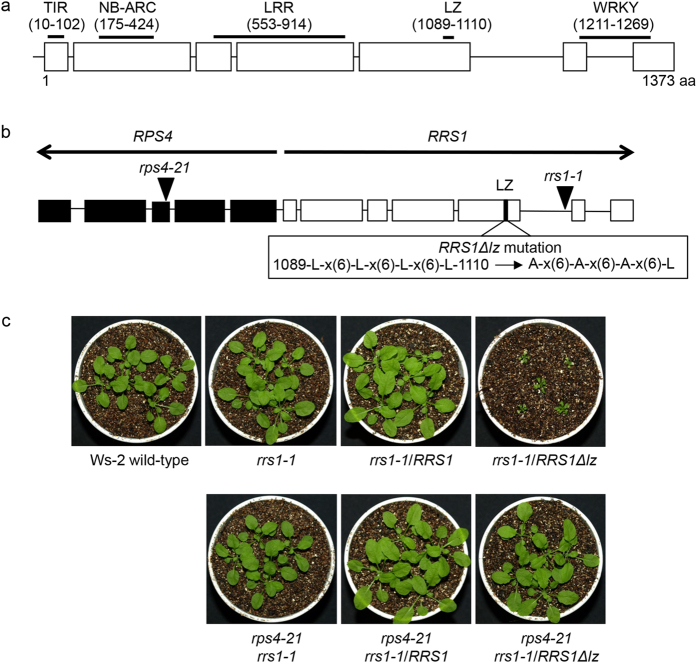
Analysis of leucine zipper (LZ) motif in RRS1. (**a**) The predicted domains and motifs in RRS1 (Ws-2 accession). Schematic gene structure of *RRS1* with exons shown as boxes and introns as lines. Localisation and amino acid positions of TIR, NB-ARC (nucleotide-binding adaptor shared with Apaf-1, plant resistance proteins, and CED-4), leucine rich repeat (LRR), LZ, nuclear localisation signal (NLS), and WRKY domains is indicated. The transformation of *Arabidopsis* mutants was performed using the 8.2-kbp genomic RRS1 fragments (from the Ws-2 accession) with or without the LZ mutation. (**b**) RRS1Δlz mutant was generated by L to A mutations in LZ motif. (**c**) Phenotypes of 4-week-old wild-type Ws-2, mutants, and transgenic plants under normal growth conditions. All mutants and transgenic plants originated from the Ws-2 accession.

RPS4 is a typical TIR-type NLR, while RRS1 is a TIR-type NLR with original structure that contains a C-terminal WRKY domain, which is a class of DNA-binding transcription factors in plants^29^. In the present study, we found a LZ motif, LRVSYDDLQEMDKVLFLYIASL, located between the LRR and WRKY domains in RRS1 (Fig. 3a). We investigated the natural sequence variation of amino acid residues across 19 *A. thaliana* accessions for which large-scale single nucleotide polymorphism (SNP) genotyping of RRS1 is possible: Bay-0, Br-0, Bur-0, C24, Col-0, Cvi-0, Est-1, Fei-0, L*er*-1, Lov-5, Nfa-8, Rrs-7, Rrs-10, Sha, Tamm-2, Ts-1, Tsu-1, Van-0, and Ws-2^30^.

The LZ motif is completely conserved in these 19 accessions, which contain both RRS1-R and RRS1-S. Saucet *et al.*^31^ had reported that RRS1B and RPS4B, similar to RRS1 and RPS4, confer recognition of AvrRps4 but not PopP2. In RRS1B from Ws-2, we also found two LZ motifs, LKGSLSSLPNVLRLLHWENYPL and LRVRYAGLQEIYKALFLYIAGL.

**Figure 4:**
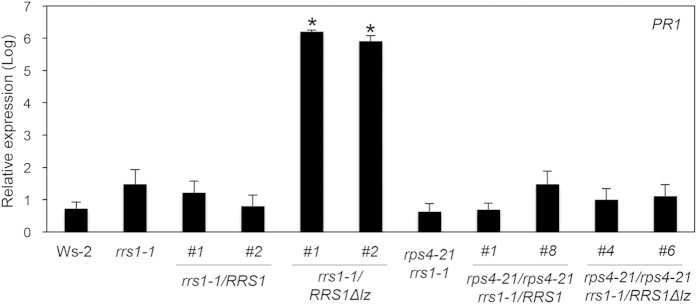
Expression of defence-related *PR1* in transgenic plants under normal growth conditions. Expression levels of *PR1* in transgenic plants under normal growth conditions were monitored by quantitative real-time PCR. The relative expression level was normalised against the expression level of *CBP20*, which is constitutively expressed. Each sample was repeated at least three times. Bars indicate the standard error (SE). The asterisks indicate significant differences compared with wild-type Ws-2 (Dunnett’s method, *P* < 0.05)^50^. The nucleotide sequence of the gene-specific primer is listed in Table S1.

The LZ is a protein–protein interaction domain consisting of an α-helical conformation with a leucine residue at every seventh position, which often facilitates dimerisation^32^. To investigate the role of the LZ motif in RRS1 (from Ws-2), we generated mutations in three leucine residues within the zipper (Fig. 3b) and named the mutated protein RRS1Δlz. We observed that the *rrs1-1/RRS1Δlz* mutants grew abnormally and constitutively expressed the inducible defence gene, *PR1*. Compared with expression in wild-type Ws-2, *PR1* gene expression was three hundred thousand- (*rrs1-1*/*RRS1Δlz*#1) and one hundred fifty thousand-fold (*rrs1-1*/*RRS1Δlz*#2) higher. Therefore, introducing *RRS1Δlz* induced autoimmune response (Figs 3c and 4). Interestingly, the *rrs1-1/rps4-21/RRS1Δlz* mutants, in which *RPS4* was absent, grew normally and did not constitutively express *PR1*, suggesting that *RPS4* was required for autoimmune response in the *rrs1-1/RRS1Δlz* mutant (Figs 3 and 4).

### Transient expression of dual R proteins induces an HR-like cell death in *N. benthamiana*

**Figure 5:**
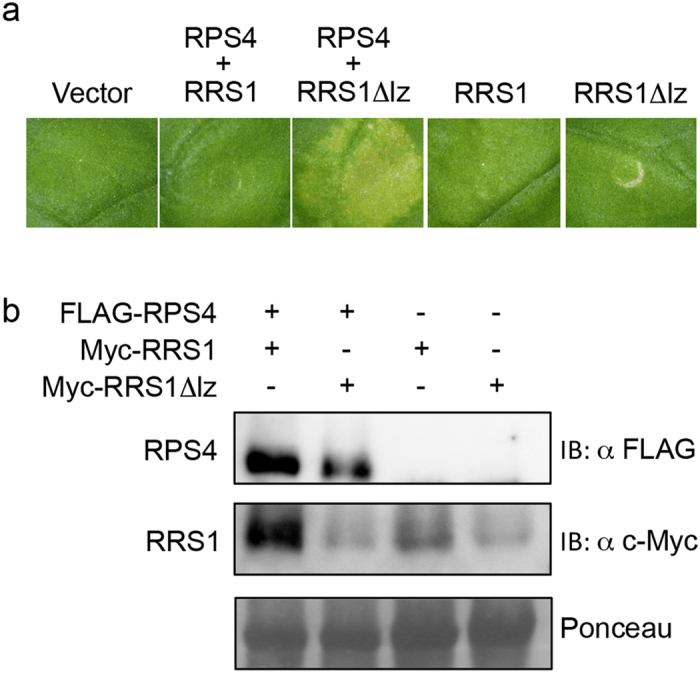
Transient expression of dual R proteins induces hypersensitive (HR)-like cell death in *Nicotiana benthamiana*. Leaves of *N. benthamiana* were infiltrated with *Agrobacterium* carrying full-length *FLAG-gRPS4*, *Myc-gRRS1*, and *Myc-gRRS1Δlz*. (**a**) HR-like cell death was observed at 3 d post-inoculation (dpi). The photograph was taken at 7 dpi under white light to visualise cell death. A set of *gRPS4* and *gRRS1Δlz* induced HR-like cell death. (**b**) Immunodetection of non-mutated RPS4, as well as RRS1 with a mutated LZ motif. Transiently co-expressed FLAG-RPS4 and Myc-RRS1 or Myc-RRS1Δlz were detected in the total protein extraction from *N. benthamiana* 44 h after *Agrobacterium* infiltration. Immunodetection was performed with either anti-FLAG or anti-Myc antibodies.

Transient expression of both full-length *RPS4* and *RRS1Δlz* induced HR-like cell death (Fig. 5a). The HR symptoms appeared 3 d after the injection of full-length *RPS4* and *RRS1Δlz* into *N. benthamiana*. However, transient expression of full-length RRS1, RRS1Δlz, or RPS4/RRS1 did not cause HR-like cell death (Fig. 5a). In addition, we used immunoblotting to verify that HR absence was not due to absence of the R proteins. Co-expressed *RRS1*/*RPS4* and *RRS1Δlz*/RPS4 yielded strong and weak signals on immunoblots, respectively (Fig. 5b). Moreover, RRS1Δlz and RRS1 were weakly detected in the total protein extraction from *RRS1Δlz*- and *RRS1*-injected *N. benthamiana* (Fig. 5b). These results confirm that the lack of HR was not due to the absence of R proteins.

### Immunoprecipitation analysis of RPS4 and RRS1Δlz

Tissue samples of *N. benthamiana* were harvested 44 h after *Agrobacterium* infiltration. The nucleus and microsomal fractions were extracted and immunoassayed with anti-FLAG and anti-Myc antibodies. Under these conditions, FLAG-RPS4 and Myc-RRS1 were detected as strong signals on immunoblotting in the microsomal fraction and as weak signals in the nucleus fraction (Fig. 1c). In contrast, RPS4 and RRS1Δlz isolated 44 h after *Agrobacterium* infiltration were weakly detected in the microsomal fraction but not in the nucleus fraction, although both mRNAs were detected in the leaves (Fig. 1c and S2).

### RPS4/RRS1Δlz-triggered cell death depends on EDS1

**Figure 6:**
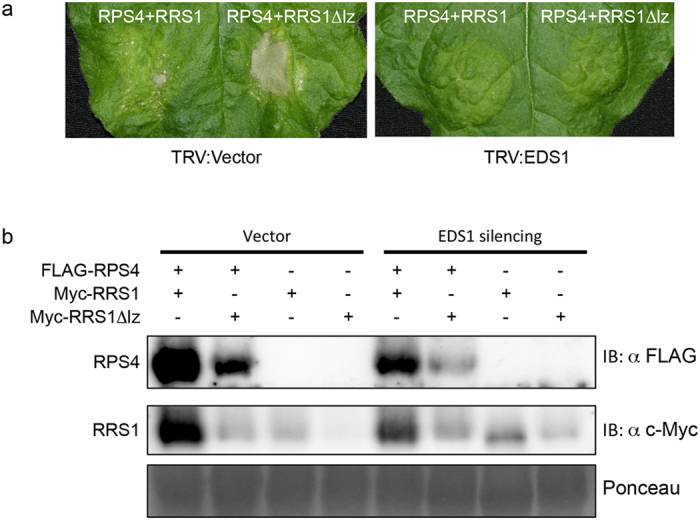
EDS1 is required for dual R protein-induced hypersensitive (HR)-like cell death. TRV:Vector or TRV:EDS1-silenced *N. benthamiana* leaves were infiltrated with *Agrobacterium* containing *FLAG-gRPS4*, and *Myc-gRRS1* or *Myc-gRRS1Δlz* constructs. (**a**) HR-like cell death was observed at 3 dpi. The photograph was taken at 7 dpi. (**b**) Immunodetection of non-mutated RPS4, as well as RRS1 with a mutated LZ motif. Total proteins extracted from *N. benthamiana* leaves 44 h after *Agrobacterium* infiltration showed the transient co-expression of FLAG-RPS4 and Myc-RRS1 or Myc-RRS1Δlz. Immunodetection was performed with either anti-FLAG or anti-Myc antibodies.

To investigate whether RPS4/RRS1Δlz-triggered cell death depends on EDS1, virus-induced gene silencing (VIGS) was used^33^. An *N. benthamiana* seedling was silenced for *NbEDS1* by inoculation with *Agrobacterium*-carrying tobacco rattle virus TRV:EDS1. The *N. benthamiana* plants that were inoculated with *Agrobacterium* harbouring TRV:GFP were used as control. Following the first inoculation, *RPS4* and *RRS1Δlz* were transiently expressed in the upper leaves of silenced *N. benthamiana* plants. The HR-like cell death phenotype was monitored at 7 d post-inoculation (dpi). The results indicated that co-expression of *RPS4* and *RRS1Δlz* conferred an HR-like cell death in the TRV:00 silenced control plants (Fig. 6a). Silencing of *NbEDS1* in *N. benthamiana* completely abolished the HR phenotype (Fig. 6a). This result indicated that VIGS of *NbEDS1* impaired HR because of *RPS4* and *RRS1Δlz* co-expression, and that *NbEDS1* was required for *RPS4/RRS1Δlz*-triggered HR in *N. benthamiana*. Using VIGS, we found that cell death resulting from the co-expression of *RPS4* and *RRS1Δlz* was due to the signalling component EDS1.

In addition, we used immunoblotting of the total protein extraction to analyse RRS1 and RRS1Δlz accumulation in both the presence and absence of RPS4 and EDS1 (Fig. 6b). With *RPS4/RRS1* co-expressed *N. benthamiana*, EDS1 absence resulted in a decrease of RRS1 and RPS4 accumulation, compared with EDS1 presence. However, RRS1Δlz was weakly detected in both the presence and absence of EDS1. Finally, with *RRS1Δlz* expressed *N. benthamiana*, RRS1Δlz was weakly detected in the absence of RPS4.

VIGS is known to reduce the level of target mRNA^34^,^35^. To investigate whether TRV:EDS1 silencing causes a reduction in *NbEDS1* mRNA, we performed mRNA expression analysis. *NbEDS1* mRNA drastically decreased in TRV:EDS1-silenced plants after the second inoculation (Fig. S3). However, *NbEDS1* mRNA was expressed in the control plants infected with TRV:GFP after the second inoculation (Fig. S3).

### Characterisation of RPS4/RRS1Δlz-triggered cell death

**Figure 7:**
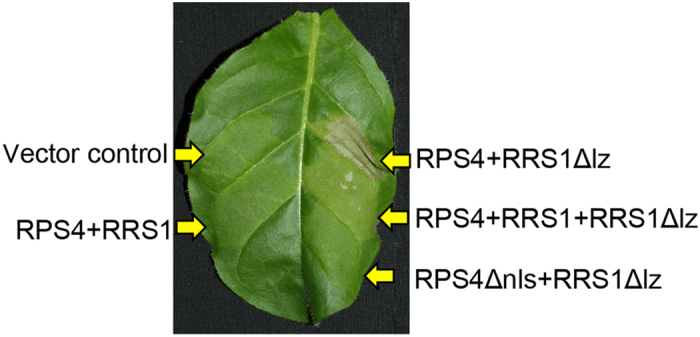
Characterisation of RPS4/RRS1Δlz-triggered cell death in *Nicotiana tabacum*. Leaves of *N. tabacum* were infiltrated with *Agrobacterium* carrying full-length *FLAG-gRPS4*, *FLAG-gRPS4Δnls*, *Myc-gRRS1*, and *Myc-gRRS1Δlz*. HR-like cell death was observed at 2 dpi, induced by sets of *gRPS4*/*gRRS1Δlz* and *gRPS4*/*gRRS1*/*gRRS1Δlz*. The photograph was taken at 4 dpi under white light to visualise cell death.

Nuclear localisation of RPS4, containing nuclear localisation sequence (NLS), is necessary for AvrRps4-triggered cell death^19^,^36^. We investigated whether HR was abolished when RRS1Δlz was co-expressed with RPS4Δnls. We found that RRS1Δlz/RPS4Δnls did not induce HR (Fig. 7). Therefore, nuclear localisation of RPS4 is necessary for RRS1Δlz-mediated cell death.

Co-expression of RRS1WT was reported to prevent RRS1-SLH1/RPS4-dependent constitutive HR, indicating that RRS1-SLH1-dependent auto-activation is recessive^36^. We also investigated whether RRS1Δlz/RPS4-dependent constitutive HR was prevented by co-expression of RRS1WT. Our results revealed that HR was weakened, but not abolished, when triggered by RRS1Δlz/RPS4/RRS1WT as opposed to RRS1Δlz/RPS4. These data suggest that RRS1WT did not completely interfere with RRS1Δlz/RPS4 triggered HR (Fig. 7). Therefore, auto-active alleles of *RRS1Δlz* are semi-dominant.

## Discussion

Dual R proteins, RPS4 and RRS1, function as a complex and mediate defence response^16^,^17^. Zhang and Gassmann^37^ previously reported that 35S:FLAG-genomicRPS4-Ler construct produced low levels of full-length protein in *N. benthamiana*. In this study, we showed that full-length N-terminally FLAG-tagged RPS4 was only slightly detected in the microsomal fraction eluted from *N. benthamiana* leaves in the absence of RRS1. In contrast, co-expressed full-length RPS4 and RRS1 gave strong signals on immunoblots, indicating that RPS4/RRS1 is stable *in planta*. Using co-immunoprecipitation assays, we found that full-length RPS4 and RRS1 strongly interact with one another *in planta*. Williams *et al.* 2014 also showed that RPS4 interacts with the RRS1 TIR domain^21^. However, co-expressed full-length RPS4 and RRS1Δlz were weakly detected in the microsomal fraction and the total protein extraction eluted from *N. benthamiana* leaves. Additionally, we verified with quantitative real-time polymerase chain reaction (qRT-PCR) that *RPS4* and *RRS1* mRNAs accumulated at similar levels in *N. benthamiana* leaves (Fig. S2).

Previous studies showed that the N-terminal NBS and LZ motif are critical for the function of NBS-LRR type R protein, RPS2^38^, and that WRKY transcription factors play an important role in plant defence^39^. WRKY 18, WRKY 40, and WRKY60 have potential LZ motifs at the N-terminus that are involved in the physical interaction of these WRKY proteins^40^. WRKYs with no LZ motifs are unable to interact with themselves and with each other. The C-terminal portion of RRS1 also possesses two conspicuous structural motifs, the LZ and the WRKY domain, that play a key role in protein–protein interaction. In this study, direct mutagenesis revealed that the C-terminal LZ motif in RRS1 is important for RPS4/RRS1 immunity. Noutoshi *et al.* also showed that a 3-bp insertion mutation of the WRKY domain in RRS1 (named RRS1-SLH1) causes autoimmunity^41^.

In this study, transient co-expression of *RPS4/RRS1Δlz* in *N. tabacum* and *N. benthamiana* induced strong HR-like cell death. However, we did not observe HR-like cell death in *N. benthamiana* induced by the transient expression of full-length *RRS1, RRS1Δlz,* or *RPS4/RRS1*. In *N. tabacum,* when transient *RPS4* expression in the absence of AvrRps4 causes HR, it is called the R gene overdose effect^19^,^42^. We also observed stunting and mimic lesions in *RPS4/RRS1Δlz*-transgenic *Arabidopsis* plants, but not in *rps4/RRS1Δlz* transgenic plants without *RPS4*. We assume that RRS1 regulates the activation of RPS4 in the absence of Avr proteins, and Avr proteins either direct or indirect modify RRS1 to activate RPS4. The structural change of RRS1 by Avr proteins is necessary for the activation of downstream defence responses. Therefore, the coexistence of RRS1 and RPS4 is essential for pathogen recognition and defence responses. On the other hand, RRS1B and RPS4B also confer recognition of AvrRps4 but not PopP2^31^. In our preliminary experiments, we found that RRS1B was not required for resistance to *C. higginsianum*.

Interestingly, we found that *Arabidopsis* mutants complemented by C-terminally tagged *RPS4-* or *RRS1*-transgenes did not confer resistance to *C. higginsianum*, whereas those complemented by N-terminally tagged *RPS4-* or *RRS1*-transgenes did. These results imply that the C-termini of both RPS4 and RRS1 play a key role in the activation of RPS4/RRS1-mediated defence responses. We previously reported that *RRS1* alleles from three *C. higginsianum-*susceptible accessions, Bur-0, Col-0, and Cvi-0, contain a premature stop codon^16^. In these accessions, the C-terminal region of RRS1 that follows the WRKY domain is relatively short compared with several resistant accessions (Ws-2 etc.). Recent reports^22^,^23^ suggest that C-terminal extension is necessary for resistance signalling, because the C-terminal region is required for PopP2 but not for AvrRps4. Thus, the C-terminal region of RRS1 likely plays a key role in the activation of RPS4/RRS1-mediated defence responses.

*EDS1* encodes a lipase-like protein that functions in R protein-mediated and basal plant disease resistance^43^. In addition, *EDS1* and *NbEDS1* are required for RPS4-mediated disease resistance in *Arabidopsis* and tobacco, respectively^42^,^44^. In present study, we found that RPS4/RRS1Δlz-triggered HR is completely abolished in *NbEDS1*-silenced *N. benthamiana* plants. Our results suggested that *RPS4/RRS1Δlz*-triggered HR also requires *NbEDS1* as a signalling component in *N. benthamiana* plants.

In conclusion, our data indicated that RPS4/RRS1 is required for active defence responses, and some structural domains that constitute these R proteins contribute to the interaction of RPS4 with RRS1.

## Methods

### Plant materials

*A. thaliana* plants were grown in soil mix (Sakata Seed Corp.) and expanded vermiculite (2–5 mm granules) at a 1:1 ratio, in a growth chamber at 22 °C under a medium-day photoperiod (12-h light/12-h night). The accessions, mutants, and transformants used in this study are of Ws-2 background. Tobacco plants (*N. benthamiana* and *N. tabacum*) were grown in soil mix and expanded vermiculite (2–5 mm granules) at a 2:1 ratio, in a growth chamber at 25 °C under a long-day photoperiod (16-h light/8-h night).

### Construction of the *R*-gene plasmid

All DNA fragments containing RRS1 and/or RPS4 used in this study derived from genome of the *A. thaliana* Ws-2 accession. Plasmids used in this study were constructed by Gateway^®^ technology following manufacturer protocol (Life Technologies, USA). All clones were verified with DNA sequencing. The pCR8GW-RR-Ws plasmid was cloned using a 10.9-kbp genomic fragment containing *RPS4* and *RRS1* as previously described^17^. The genomic fragment of *RRS1* (coding region) was PCR-amplified using pCR8GW-RR-Ws as template and cloned into pCR8GW-TOPO (named pCR8GW-gRRS1). To generate the destination vector pGWB18Ω (35S:Ω:4xMyc), the *Hin*dIII and *Xba*I regions in pGWB18 were replaced with 35S enhancers and the omega leader sequence cassette in pBE2113^45^. To create the destination vector pGWB18-RRS1p, the 35S promoter region in pGWB18 was replaced with the *RRS1* promoter region (1.8 kbp upstream of the start codon) using the In-Fusion^®^ HD Cloning Kit (Takara Bio Inc., Japan). To generate the 35S:Ω:4 × Myc-gRRS1 and RRS1p:4 × Myc-gRRS1 constructs, LR reactions were performed to recombine the entry clone, containing genomic *RRS1*, into the Gateway^®^-compatible destination vectors: pGWB18Ω for *Agrobacterium*-mediated transient expression and pGWB18-RRS1p for stable *Arabidopsis* transformation *via* LR reaction. Site-directed mutagenesis of genomic *RRS1* with or without the promoter region^16^ was performed by a custom cloning service (Takara Bio Inc., Japan) to generate *RRS1Δlz* carrying Leu to Ala mutations (L1089A, L1096A, and L1103A) at the LZ motif. Subsequently, LR cloning was used to generate binary constructs in pGWB18Ω for *Agrobacterium*-mediated transient expression and in pGWB1 for stable *Arabidopsis* transformation.

The 6.3-kbp genomic *RPS4* fragment, including approximately 2.1-kbp upstream and 109-bp downstream regions, was cloned into pCR8GW-TOPO (named pCR8GW- RPS4p:gRPS4). To generate the RPS4p:3 × FLAG-gRPS4 construct, synthetic 3 × FLAG and a spacer sequence, MDYKDHDGDYKDHDIDYKDDDDKGGGS, was inserted just before the *RPS4* start codon by a custom cloning service (Life Technologies, USA). To generate the pCR8GW-35S:Ω:3 × FLAG-gRPS4, the *RPS4* promoter region in pCR8GW-RPS4p:gRPS4 was replaced by 35S enhancers and the omega leader sequence cassette in pBE2113, using the In-Fusion^®^ HD Cloning Kit (Takara Bio Inc., Japan). To create the destination vector pBGYNΔEYFP-nuc, the EYFP-nuc cassette in pBGYN (Inplanta Innovations Inc., Japan) was cut with *Sma*I and *Sac*I. The *attR2* region in the Gateway^®^ cassette was engineered with PCR and inserted into the vector using the In-Fusion^®^ HD Cloning Kit (Takara Bio Inc., Japan). RPS4p:3 × FLAG-gRPS4 and 35S:Ω:3 × FLAG-gRPS4 were recombined into the destination vectors, pBGYNΔEYFP-nuc, for stable *Arabidopsis* transformation and pGWB1 for *Agrobacterium*-mediated transient expression *via* LR reaction. The DNA fragment of 35S: Ω:3 × FLAG-gRPS4-TIR (encoding the N-terminal amino acids 1–235) was PCR-amplified using pGWB1-35S:Ω:3 × FLAG-gRPS4 as a template and cloned into pCR8GW-TOPO. The 35S:Ω:3 × FLAG-gRPS4-TIR construct was sub-cloned into the destination vector pGWB1 *via* LR reaction.

### *Arabidopsis* transformation

*Arabidopsis* transformation was carried out according to the floral inoculating method using *Agrobacterium tumefaciens* strain GV3101 (pMP90)^46^.

### *C. higginsianum* inoculation and quantification of *C. higginsianum* actin mRNA

*C. higginsianum* Saccardo isolates (MAFF305635) were obtained from the Ministry of Agriculture, Forestry and Fisheries (MAFF) Genebank, Japan. *Arabidopsis* plants were inoculated as described previously^47^ and harvested at 5 dpi for qRT-PCR analysis. The quantification of *C. higginsianum* was performed as described previously^47^.

### Transient expression assay in *N. benthamiana* and *N. tabacum*

*N. benthamiana* and *N. tabacum* plants were grown in a growth chamber at 25 °C under a long-day photoperiod (16-h light/8-h night). *Nicotiana benthamiana* (32-day-old) and *N. tabacum* (32-day-old) plants were used in the experiment. Overnight bacterial cultures of *A. tumefaciens* strain GV3101 (pMP90) containing constructs with epitope-tags were harvested with centrifugation. Cells were washed three times in an induction buffer (10 mM 2-(*N*-morpholino)ethanesulfonic acid, pH 5.6, 10 mM MgCl_2_, and 150 μM acetosyringone), re-suspended in an induction buffer, and incubated for 2–4 h in darkness at 25 °C. For transient expression experiments, bacterial strains containing the constructs were mixed, adjusting each strain concentration to a final optical density of 0.5 at 600 nm (OD_600_). *Agrobacterium* cells were hand-infiltrated into *N. benthamiana* and *N. tabacum* leaves with a 1-mL blunt-end syringe. Cell death responses began to appear on leaves 3 and 2 d after *Agrobacterium* infiltration in *N. benthamiana* and *N. tabacum*, respectively. Symptoms were documented under white light to visualise cell death.

### Protein fractionation and immunoblotting

Microsomal and soluble fractions were prepared as described previously^28^. Extraction buffer (50 mM 4-(2-hydroxyethyl)-1-piperazineethanesulfonic acid, pH 7.5, 250 mM sucrose, 15 mM ethylenediaminetetraacetic acid, 5% glycerol, 0.5% polyvinylpyrrolidone, 3 mM dithiothreitol, 2× Roche protease-inhibitor cocktail and 1× Roche phosphatase-inhibitor cocktail) (Roche, Switzerland) was added to the plant materials, which was ground with mortar and pestle, then with a Potter-Elvehjem tissue grinder. The extracts were filtered through two layers of miracloth pre-wetted with extraction buffer and centrifuged at 2,000 × *g* for 10 min at 4 °C. The supernatant consisting of the cytoplasmic fraction was further subjected to ultracentrifugation at 100,000 × *g* to separate the soluble and microsomal (pellet) fractions. The pellet was resuspended in extraction buffer containing 0.5% Igepal CA-630 (Sigma-Aldrich, USA).

Nuclear extracts were prepared with a semi-pure preparation method using the CelLytic^TM^ PN Isolation/Extraction Kit (Sigma-Aldrich, USA) and following manufacturer protocol, with one modification: 1× Roche phosphatase-inhibitor cocktail was added into NIBA buffer.

Total proteins were extracted according to previously described methods^23^.

Total protein was separated on a 4–15% sodium dodecyl sulfate polyacrylamide gel (BioRad, USA) and transferred onto a polyvinylidene difluoride membrane. Immunoblots were performed with monoclonal anti-FLAG (Sigma-Aldrich, USA) and anti-c-Myc (Roche, Switzerland) antibodies, as well as a secondary HRP-conjugated anti-mouse antibody (Promega, USA), then visualised with chemiluminescence (ECL; Bio-Rad, USA). The degree of enrichment in cellular fractionation was determined by immunoblot analyses with anti-GAPDH (Genscript, USA), anti-BiP (Agrisera, Sweden), and anti-histone H3 (Abcam, USA) antibodies. Secondary HRP-conjugated anti-mouse, anti-goat, and anti-rabbit antibodies (Promega, USA) were used for detecting chemiluminescent immunoblots.

The co-immunoprecipitation assays were performed using Anti-FLAG M2 affinity and anti-c-Myc affinity gels (Sigma, USA) following manufacturer protocol. The protein concentrations of fractions were determined with Bradford assays^48^ using bovine serum albumin as a standard. Protein samples (1 mg) of microsomal fractions were incubated with Anti-FLAG M2 for 2 h or anti-c-Myc for 1 h affinity gels (Sigma-Aldrich, USA) at 4 °C. The immunoprecipitates were analysed with immunoblotting using monoclonal anti-FLAG (Sigma-Aldrich, USA) and anti-c-Myc (Roche, Switzerland) antibodies. A special secondary antibody (Mouse TrueBlot anti-Mouse Ig HRP; Rockland, USA) that only detects full-length immunoglobulin was used for immunoblotting to avoid any nonspecific bands.

### VIGS

Using a tobacco rattle virus vector, VIGS of the *N. benthamiana* homologue *EDS1* was performed as described previously^49^. *Nicotiana benthamiana* plants (14-day-old) were infiltrated with *Agrobacteria* carrying TRV:EDS1 to silence *NbEDS1*. After 3 to 4 weeks, the upper leaves of these plants were used for an *Agrobacterium*-mediated transient assay.

## Additional Information

**How to cite this article**: Narusaka, M. *et al.* Leucine zipper motif in RRS1 is crucial for the regulation of *Arabidopsis* dual resistance protein complex RPS4/RRS1. *Sci. Rep.* **6**, 18702; doi: 10.1038/srep18702 (2016).

## Supplementary Material

Supplementary Information
