# Supplementary material for: Leucine zipper motif in RRS1 is crucial for the regulation of Arabidopsis dual resistance protein complex RPS4/RRS1
Source: Sci Rep. 2016 Jan 11;6:18702. doi: 10.1038/srep18702 (PMC4707544; doi:10.1038/srep18702)
Supplement: Supplementary Information [file srep18702-s1.pdf]

Supplementary Table, Figures, and Figure Legends

Leucine zipper motif in RRS1 is crucial for the regulation of *Arabidopsis* dual resistance protein complex RPS4/RRS1

Mari Narusaka[1], Kazuhiro Toyoda[2], Tomonori Shiraishi[1], Satoshi Iuchi[3], Yoshitaka Takano[4], Ken Shirasu[5], and Yoshihiro Narusaka[1*]

[1]Research Institute for Biological Sciences Okayama, Okayama 716-1241, Japan

[2]Faculty of Agriculture, Okayama University, Okayama 700-8530, Japan

[3]RIKEN BioResource Centre, Tsukuba 305-0074, Japan

[4]Graduate School of Agriculture, Kyoto University, Kyoto 606-8502, Japan

[5]RIKEN Centre for Sustainable Resource Science, Yokohama 230-0045, Japan

[*]Correspondence to:

Yoshihiro Narusaka, Research Institute for Biological Sciences Okayama, 7549-1 Yoshikawa, Kibityuo-town, Kaga-gun, Okayama 716-1241, Japan; Tel: +81-866-56-9450; Fax: +81-866-56-9453; E-mail: yo_narusaka@bio-ribs.com

Supplementary Table S1. Quantitative real-time PCR primers used in this study.

	Forward primer	Reverse primer
At-CBP20	TGTTTCGTCCTGTTCTACTC	CCCATTGTCTTCCTTCTTG
AtPR1	CCCACAAGATTATCTAAGGGTTCAC	CCCTCTCGTCCCACTGCAT
NtEF1α	TCTGTTGAGATGCACCACGAAG	ACAAACCCACGCTTGAGATCC
RPS4	GTGACAAGGACACACCATGC	GCTTTCAGTGTTGTGCAACCTTC
RRS1	GTTCTCGTTTTCCAAGGGGTTA	CTAACATGTTTGAATCGGTCTCG
NtEDS1	GCTTGTAACTTAGCAATGGAAGCTC	GCTACCTCATCTGTGCCAACAC
Ch-ACT	CTCGTTATCGACAATGGTTC	GAGTCCTTCTGGCCCATAC

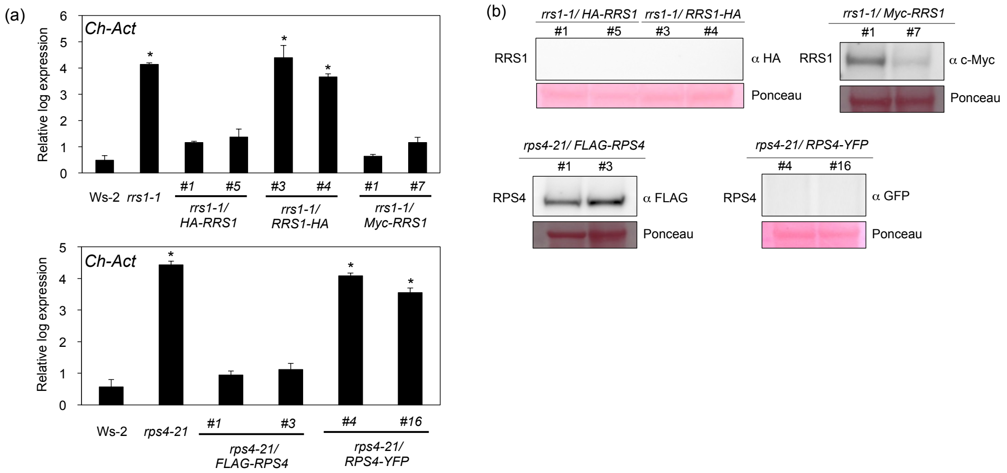

Supplementary Figure S1. Tag-fused RPS4 and RRS1 confer resistance against *Colletotrichum higginsianum*. We cloned PCR products to generate native promoter-*HA-gRRS1*, native promoter–*gRRS1-HA*, and native promoter-*Myc-gRRS1* for stable *Arabidopsis* transformation. We cloned PCR products to generate native promoter-*FLAG-gRPS4* and native promoter-*gRPS4-YFP* for stable *Arabidopsis* transformation. (a) Transgenic plants with N- or C-terminal tagged RPS4 or RRS1 grown for 28–30 d (12-h light/12-h dark photoperiod) were sprayed with a spore suspension (5×10^5 spores ml^{-1}). Leaves were harvested at 5 d post inoculation, and total RNA was isolated. Quantitative real-time PCR was performed for each sample using *Ch-ACT* and *At-CBP20* primer pairs. The experiment was repeated three times. Bars indicate standard errors (SE). Asterisks indicate significant differences compared to Ws-2 (Dunnett's method, $P < 0.05$) (50). The C-terminus of RPS4 and RRS1 is responsible for *C. higginsianum* resistance in *A. thaliana*. (b) Immunodetection of microsomal extracts from transgenic plants with N- or C-terminal tagged RPS4 or RRS1 leaves. With immunoblotting, N-terminal tagged 4×Myc-RRS1 and 3×FLAG-RPS4 were detected in transgenic *Arabidopsis*, but the transgenic plants with C-terminal tagged RRS1-3×HA and RPS4-YFP, as well as N-terminal tagged 3×HA-RRS1, were not detected.

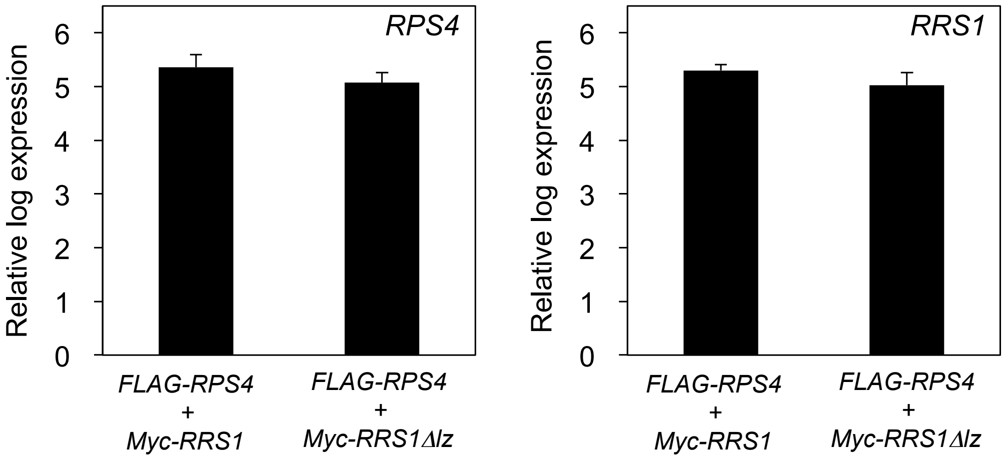

Supplementary Figure S2. Expression analysis of *FLAG-RPS4*, *Myc-RRS1*, and *Myc-RRS1Δlz* in *Agrobacterium*-infiltrated *Nicotiana benthamiana* leaves. To investigate whether expression of *FLAG-RPS4*, *Myc-RRS1*, and *Myc-RRS1Δlz* were induced in *N. benthamiana* tissue samples harvested 44 h after *Agrobacterium* infiltration (Fig. 1c), we performed mRNA expression analysis. Expression levels of these tag-fused genes were monitored by quantitative real-time PCR. The relative expression level was normalised against the expression level of *NtEF1α*, which is constitutively expressed in *N. benthamiana* leaves. Each sample was repeated at least two times. Bars indicate the standard errors (SE). No significant differences were identified among the samples (Dunnett's method, $P < 0.05$) (50). The nucleotide sequence of the gene-specific primer is listed in Table S1.

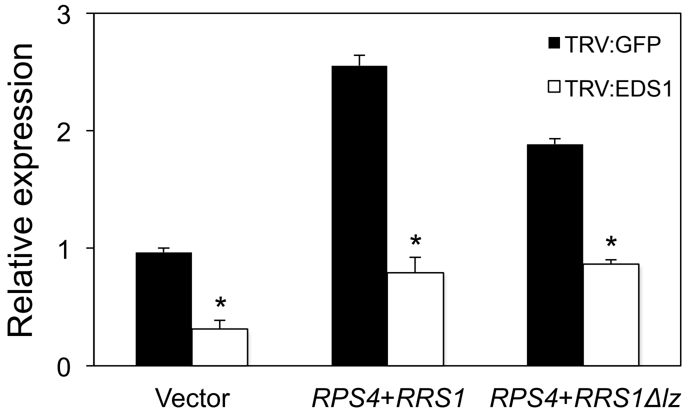

Supplementary Figure S3. Reduction of *NbEDS1* mRNA caused by TRV:EDS1 silencing. To investigate whether TRV:EDS1 silencing caused a reduction of *NbEDS1* mRNA, we performed mRNA expression analysis. Expression levels of *NbEDS1* mRNA were monitored by quantitative real-time PCR. The relative expression level was normalised against the expression level of *NtEF1α*, which is constitutively expressed in *Nicotiana benthamiana* leaves. Each sample was repeated at least two times. Bars indicate the standard error (SE). Asterisks indicate significant differences compared to the controls (Dunnett's method, $P < 0.05$) (50). The nucleotide sequence of the gene-specific primer is listed in Table S1.
